# A neurodevelopmental TUBB2B β-tubulin mutation impairs Bim1 (yeast EB1)-dependent spindle positioning

**DOI:** 10.1242/bio.038620

**Published:** 2019-01-23

**Authors:** Eric Denarier, Carine Brousse, Abdoulaye Sissoko, Annie Andrieux, Cécile Boscheron

**Affiliations:** 1 Univ. Grenoble Alpes, Grenoble Institut des Neurosciences, GIN, F-38000, Grenoble, France; 2 Institut National de la Santé et de la Recherche Médicale (INSERM), U1216, F-38000, Grenoble, France; 3 Commissariat à l'Energie Atomique et aux Energies Alternatives (CEA), Biosciences and Biotechnology Institute of Grenoble, Grenoble, France; 4 Institut National de la Transfusion Sanguine (INTS), F-75015 Paris, France; 5 Paris Descartes University, F-75006 Paris, France; 6 Institut de Biologie Structurale (IBS) , F-38000 Grenoble, France

**Keywords:** EB1, Microtubule, Spindle positioning, TUBB2B, Yeast

## Abstract

Malformations of the human cerebral cortex can be caused by mutations in tubulins that associate to compose microtubules. Cerebral cortical folding relies on neuronal migration and on progenitor proliferation partly dictated by microtubule-dependent mitotic spindle positioning. A single amino acid change, F265L, in the conserved TUBB2B β-tubulin gene has been identified in patients with abnormal cortex formation. A caveat for studying this mutation in mammalian cells is that nine genes encode β-tubulin in human. Here, we generate a yeast strain expressing F265L tubulin mutant as the sole source of β-tubulin. The F265L mutation does not preclude expression of a stable β-tubulin protein which is incorporated into microtubules. However, impaired cell growth was observed at high temperatures along with altered microtubule dynamics and stability. In addition, F265L mutation produces a highly specific mitotic spindle positioning defect related to Bim1 (yeast EB1) dysfunction. Indeed, F265L cells display an abnormal Bim1 recruitment profile at microtubule plus-ends. These results indicate that the F265L β-tubulin mutation affects microtubule plus-end complexes known to be important for microtubule dynamics and for microtubule function during mitotic spindle positioning.

## Abstract (precis)

**Summary:** Patients with intellectual disabilities carry a F265L mutation in TUBB2B β-tubulin gene. Yeast used as a cellular model reveals spindle mis-positioning associated with reduced yeast EB1 affinity for microtubule plus-ends.

## INTRODUCTION

Malformation in cortical development (MCD) describes a group of severe brain malformations associated with intellectual disability and refractory infantile epilepsy. MCDs include polymicrogyria, in which an excessive number of abnormally small gyri are found in the cerebral cortex. Recently, patients with polymicrogyria associated with severe mental retardation and epileptic seizures were shown to carry a single *de novo* heterozygous amino-acid substitution: a phenylalanine to leucine mutation at position 265 in the conserved β-tubulin TUBB2B gene (Bahi-Buisson et al., 2014; Jaglin et al., 2009).

αβ-tubulin dimers associate to compose microtubules that display dynamicity and undergo stochastic switches between growth and shrinkage phases, the hallmark phenomenon known as dynamic instability (Alushin et al., 2014; Mitchison and Kirschner, 1984; Mitchison, 2014). This remarkable feature primarily depends on the ability of tubulins to bind and hydrolyze GTP. Microtubule elongation occurs through tubulin dimer assembling at the end of the microtubule which is capped by β-tubulin subunits – dubbed ‘plus-end’ and sometimes written ‘+end’. At growing microtubule plus-ends, GTP hydrolysis is thought to be delayed with respect to tubulin polymerization, giving rise to a protective layer of GTP-tubulin dimers, the so-called ‘GTP cap’ (Carlier et al., 1984; Dimitrov et al., 2008; Pantaloni and Carlier, 1986). The GTP cap is recognized by a subclass of proteins known as ‘plus-end tracking proteins’ (+Tips) (de Forges et al., 2016; Duellberg et al., 2016; Maurer et al., 2012). +Tips play a key role in regulating microtubule dynamics, along with numerous variables including tubulin isoforms, the amount of free αβ-tubulin dimers, molecular motors and microtubule-associated proteins (Estrem et al., 2017; Lundin et al., 2010; van de Willige et al., 2016; Vemu et al., 2017).

Several microtubule-dependent processes have been implicated in the normal folding of the six-layered human cortex. Neuronal differentiation from the neural progenitor pool depends on the orientation of the division plate, which is either aligned with or perpendicular to the ventricles, as dictated by the position of the mitotic spindle (Willardsen and Link, 2011). Later, neuronal migration involves nuclear motion (Bertipaglia et al., 2017). Spindle positioning and cell migration both universally depend on (1) dynein molecules found at microtubule plus-ends and in the cell cortex that walk along and exert force on microtubules through characteristic motor activity and (2) the actin cytoskeleton and its interaction with microtubules, as mediated by linker proteins (Coles and Bradke, 2015; di Pietro et al., 2016; Howard and Garzon-Coral, 2017).

*Saccharomyces cerevisiae* was one of the first organisms where the mechanisms and active components involved in controlling mitotic spindle positioning were identified before recognizing a startling conservation of the spindle orientation mechanisms and key protein partners in microtubule function between humans and budding yeast (Andrieux et al., 2017; Siller and Doe, 2009). In yeast, mitotic spindle positioning and orientation is controlled by two pathways which were identified through studies of spindle positioning relying on yeast genetics (Miller and Rose, 1998). The first pathway involves actin/Kar9 in a microtubule-guidance mechanism occurring during the S phase of the cell cycle (Lee et al., 2000; Yin et al., 2000). Kar9 links microtubules to polarized cortical actin cables by interacting with myosin-V motor and Bim1, the yeast counterpart of the +Tips protein EB1. Thus, microtubules are guided and pulled along actin cables toward the bud by the myosin-V motor (Beach et al., 2000; Hwang et al., 2003; Lee et al., 2000), resulting in spindle alignment with the mother-bud polarity axis. The second pathway involves dynein motors which power spindle movement through the mother-bud junction. This movement initially involves dynein transportation to the tips of microtubules thanks to the +Tips Bik1 (yeast CLIP170) (Carvalho et al., 2004; Caudron et al., 2008), it is then offloaded and activated at the bud cell cortex (Lammers and Markus, 2015; Sheeman et al., 2003), where it then drags the nucleus into the bud cell (Moore et al., 2009; Yeh et al., 2000).

A number of questions are raised by the discovery of the correlation between the F265L heterozygous mutation in the TUBB2B tubulin gene and a severe neurodevelopmental disorder. Is the mutant tubulin stable and incorporated into microtubules? If it is incorporated into microtubules, does it induce changes to microtubule dynamics and/or alter binding of microtubule partners? In mammalian cells, due to the large number of tubulin isotypes, it is difficult to distinguish between these possibilities and to decipher the molecular defects arising from this mutation. Indeed, in humans, each cell β-tubulin content not only results from the heterozygous expression of wild-type (wt) and F265L TUBB2B alleles, but also from the expression of other β-tubulin genes among the nine alleles present in the human genome (Findeisen et al., 2014; Khodiyar et al., 2007). Lower eukaryotes have a smaller number of tubulin genes than vertebrates; for example, the budding yeast has two α-tubulin genes (*TUB1*, *TUB3*) and only one β-tubulin gene (*TUB2*), and this last is highly conserved (Ludueña, 1993; Schatz et al., 1986; Thomas et al., 1985). Therefore, to gain insight into the role played by the F265 residue in β-tubulin, we produced *S. cerevisiae* yeast strains mutated on F265 in *TUB2* and assessed the consequences of expressing this mutation as the sole source of β-tubulin. In mutant cells, mitosis and spindle orientation were impaired, and microtubule dynamics was altered. Furthermore, evidence of a reduced association of Bim1 (yeast EB1) with microtubule plus-ends was found. These results indicate that F265 in β-tubulin is essential for normal microtubule dynamics, cell division and Bim1 association with microtubule tips.

## RESULTS AND DISCUSSION

### Transposition of the human *F265L* mutation to yeast β-tubulin is non-lethal

**Fig. 1.**
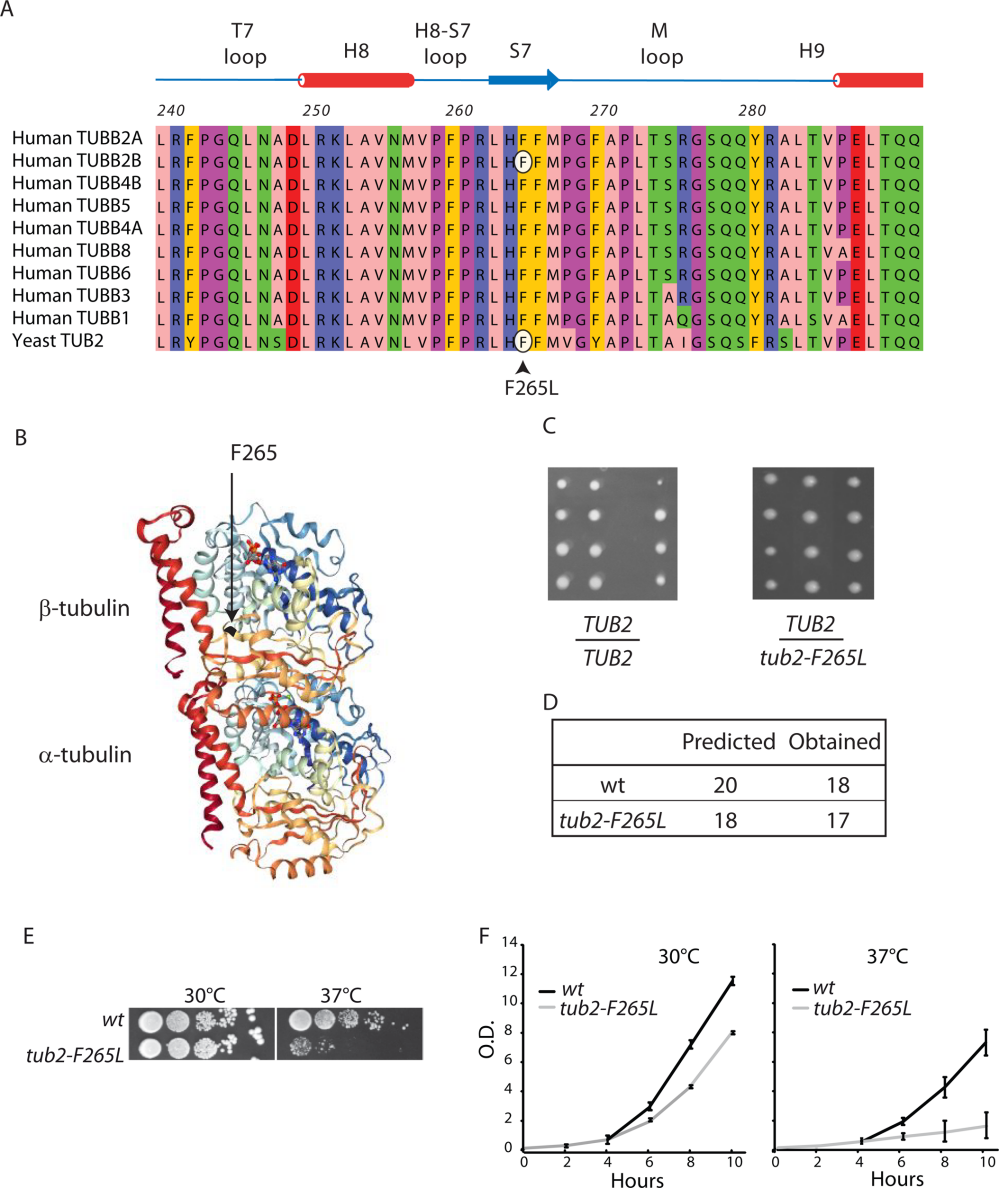
**F265L mutation in yeast β-tubulin is non-lethal.** (A) Partial protein sequence of nine human β-tubulin isotypes protein aligned with *S. cerevisiae* Tub2 protein as indicated (colored according to Zappo color code in Jalview). β-tubulin secondary structure is shown above the alignment (PDB: 5JCO). (B) Structure of the αβ-tubulin heterodimer viewed from the side (PDB: 1JFF). α-helices H11 and H12, exposed on the microtubule surface, are colored red. The arrow indicates β-tubulin residue 265 (phenylalanine). (C) Diploid wt (*TUB2*/*TUB2*) or heterozygous *tub2-F265L* (*TUB2*/*tub2-F265L*) cells were sporulated and dissected. An image of a representative portion of tetrad analysis used to assess the viability is shown. (D) Haploid *tub2-F265L* spores were viable and recovered at the expected frequency. (E) Growth at 30°C and 37°C of sequential dilutions of wt and *tub2-F265L* cells spotted on YPD media. (F) Optical density (O.D.) measured at 600 nm for liquid cultures of three separate clones of wt and *tub2-F265L* cells at 30°C or 37°C. Values correspond to mean±s.d. (*n*=3).

The sequence and protein structure of β-tubulin is highly conserved between the different human isotypes (from 99.55% identity between TUBB2A and TUBB2B to 77.25% between TUBB1 and TUBB8) and across species (Ludueña, 1993). The TUB2B Phenylalanine 265 residue is mutated to leucine in a human brain disease (Bahi-Buisson et al., 2014; Jaglin et al., 2009). This isotype of tubulin shares considerable homology with the single β-tubulin present in budding yeast (Tub2) (73.3% identity and 87.52% homology), including conservation of F265 (Fig. 1A). This residue belongs to strand S7 in the so-called intermediate domain, residing between the amino-terminal domain containing the nucleotide-binding region and the carboxy-terminal domain, which constitutes the binding surface for MAPs and motor proteins (Alushin et al., 2014; Nogales et al., 1998) (Fig. 1B).

We chose to introduce the F265L mutation into the corresponding conserved site in the yeast *TUB2* β-tubulin gene (generating yeast *tub2-F265L* strain) as replacement of yeast tubulin by human tubulin results in cell death (Sirajuddin et al., 2014). The *tub2-F265L* mutant replaced the endogenous *TUB2* gene, leaving the upstream and downstream regulatory elements intact. Heterozygous diploids containing a single mutated *TUB2* allele (*tub2-F265L*/*TUB2*) displayed no growth defects on rich media (data not shown). Sporulation and tetrad dissection revealed that haploid *tub2-F265L* spores were viable and recovered at the expected frequency (Fig. 1C,D), establishing that the *tub2-F265L* mutation produced viable cells even when present as the sole copy of β-tubulin.

We examined cell growth in *tub2-F265L* and wt strains at various temperatures by spotting cells on rich plates (Fig. 1E). Similar growth was observed for both strains at 30°C, but cell growth was substantially impaired in the *tub2-F265L* strain at 37°C. Similar results were obtained with liquid cultures, where doubling time at 37°C was increased (from 1 h 40 min for wt to 2 h 10 min for F265L) (Fig. 1F). These results indicate thermo-sensitive impairment of cell growth in *tub2-F265L* strain.

### *F265L* β-tubulin proteins are incorporated into microtubules and alter microtubule dynamics and stability *in vivo*

**Fig. 2.**
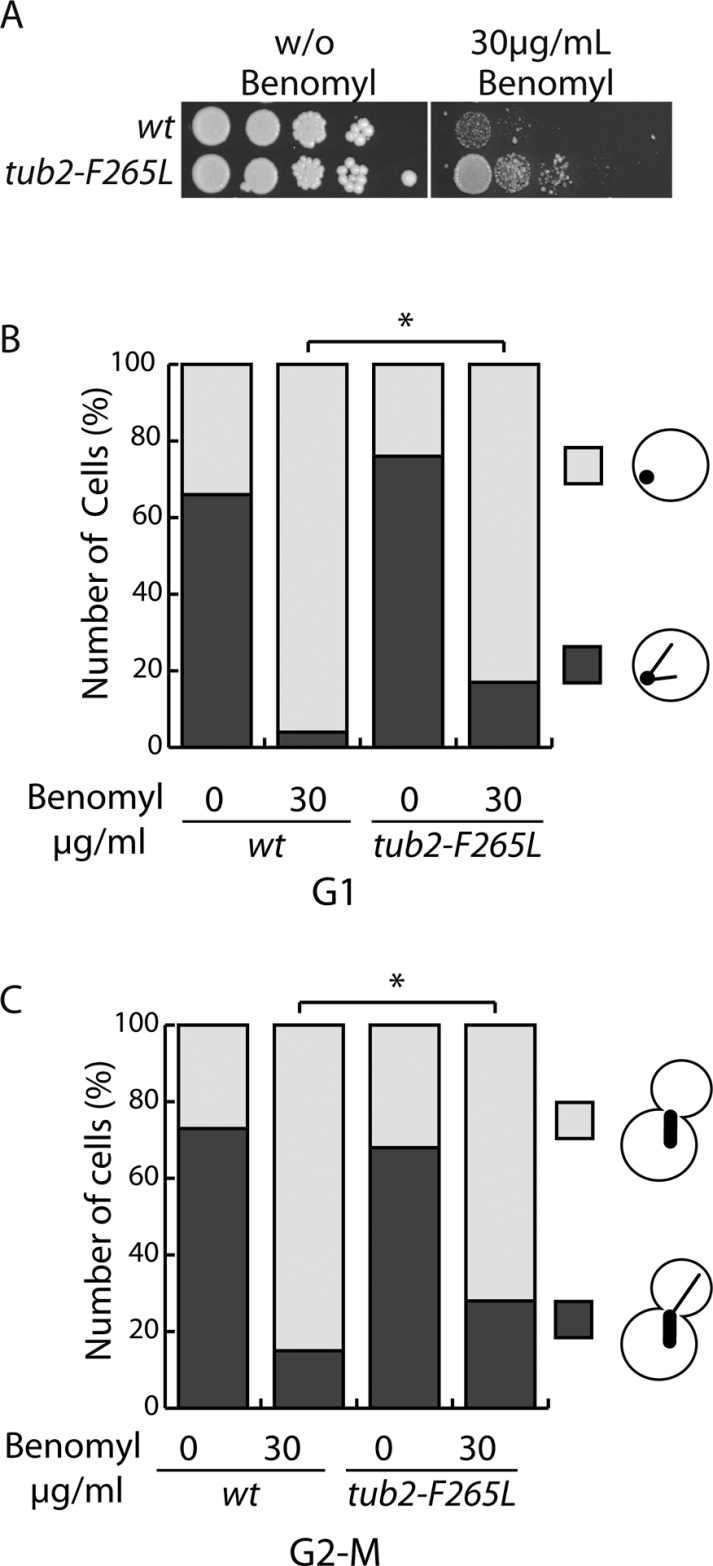
***tub2-F265L* cells have microtubules resistant to benomyl.** (A) Growth of sequential dilutions of wt and *tub2-F265L* cells spotted on YPD media without (w/o) benomyl or containing 30 µg/ml benomyl, as indicated. (B,C) Percentage of cells presenting at least one microtubule signal after liquid culture in media with or without 30 µg/ml benomyl in G1 cells (B) or G2-M cells (C). Chi-square test: *P<0.05; *n*=100.

We took advantage of the *tub2-F265L* cells' viability to observe microtubules in the *tub2-F265L* strain. Cytoplasmic microtubules were observed in live cells expressing GFP-Bik1 (yeast CLIP170) under the control of the Bik1 promoter. Initial observations of cytoplasmic microtubules in wt and *tub2-F265L* strains showed a reduced size of *tub2-F265L* microtubules and a comparable numbers in both strains (Table 1). Then, microtubule dynamics were measured in video-microscopy experiments, where the most dramatic differences between wt and *tub2-F265L* cells were observed during the period after spindle assembly and before anaphase onset (hereafter called G2-M). During this phase, the catastrophe frequency in *tub2-F265L* cells was reduced compared to wt cells (0.007/s in tub2-F265L versus 0.012/s in wt, *P*<0.05, Table 1). In addition, microtubules in *tub2-F265L* cells exhibited a marked increase in time spent in pause (59.5% versus 25.7% in wt cells, Table 1). This increased pausing was mostly accounted for by a decrease in the time spent growing (20.6% in *tub2-F265L* cells compared to 43.2% in wt cells). Longer pauses were also observed with *tub2-F265L* cells during the G1 phase (0.71 min in tub2-F265L compared to 0.49 min in wt, *P*<0.05, Table 1). Other G1 dynamics parameters were similar between *tub2-F265L* and wt. Consequently, microtubule dynamics are altered in the *tub2-F265L* strain, mainly during G2-M. The increased time in pause and decreased catastrophe frequency suggested that microtubules are more stable in the mutant strain. To investigate this point we analyzed cell growth and microtubule behavior after benomyl treatment, a drug that depolymerizes microtubules. Sensitivity and growth of the wt and *tub2-F265L* cells to benomyl were tested by spotting cells onto rich plates free from or containing the drug (up to 30 µg/ml) (Fig. 2A). In the presence of benomyl, cell growth was less impaired in *tub2-F265L* compared to wt cells, indicating super resistance to benomyl in cells expressing F265L tubulin. To check if this resistance can be the result of microtubule resistance to depolymerisation, we counted the number of cells with or without microtubule(s) after benomyl treatment (Fig. 2B,C). We observed more cells with microtubules in the *tub2-F265L* than in wt strain both in G1 (17 versus 4%) or G2-M (28 versus 15%) suggesting that their tolerance to benomyl may be due to the stability of their microtubules against depolymerization.

**Table 1.**
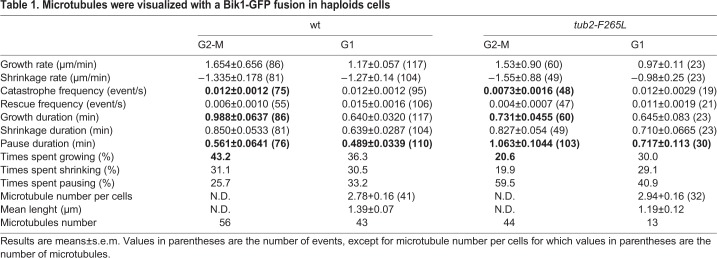
**Microtubules were visualized with a Bik1-GFP fusion in haploids cells**

Altogether, our results indicate that the F265L β-tubulin is a functional protein that can be incorporated into microtubules resulting in the decrease of their dynamics and a greater stability.

### F265L mutation causes spindle positioning deficiency

**Fig. 3.**
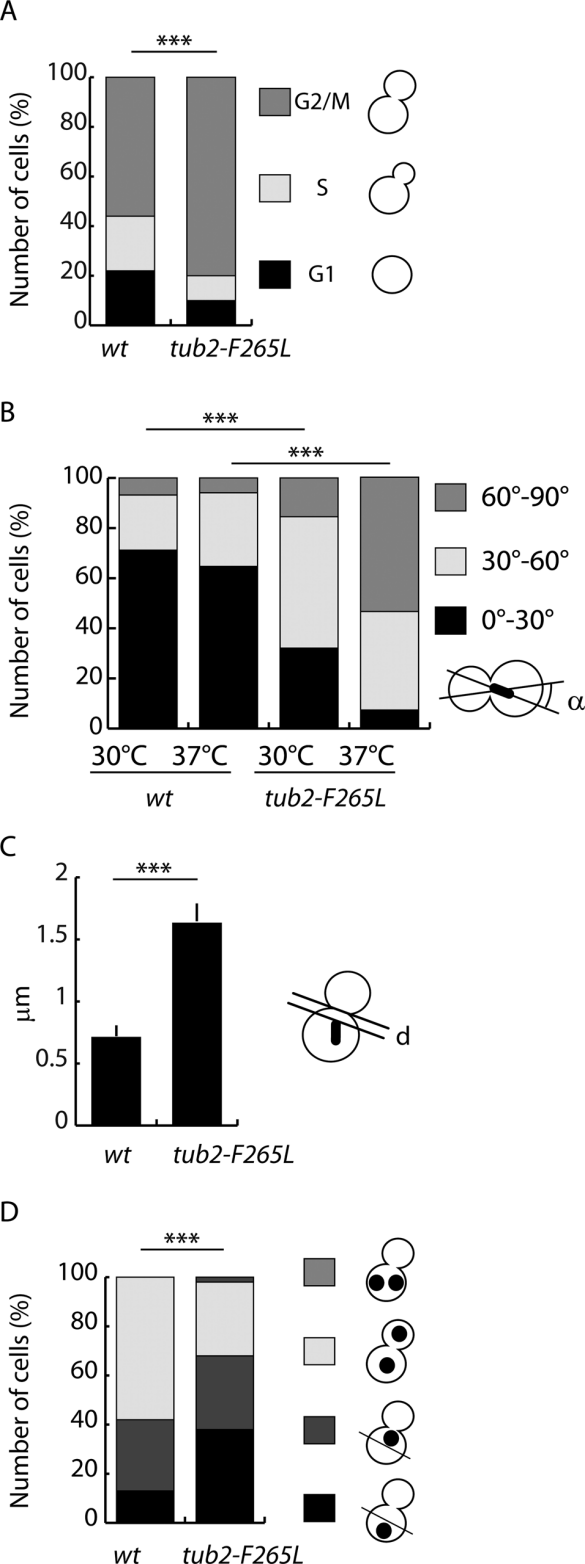
**Mitotic delay and mitotic spindle misorientation in *tub2-F265L* strain.** (A) Mitotic delay in *tub2-F265L* cells. The proportion of unbudded cells (G1), cells with small buds (S) and cells with large buds (G2/M) were scored in wt and *tub2-F265L* strains at both 30°C and 37°C, as indicated. Chi-square test: ***P<0.001; *n*>100. (B) Histograms representing the frequency of cells with bipolar spindles that are oriented between 0–30, 30–60 and >60–90 degrees relative to the mother–daughter axis of wt and *tub2-F265L* strains at 30°C and 37°C, as indicated. Chi-square test: ***P<0.001; *n*>30. (C) Distance between the neck and the neck-proximal spindle pole at 30°C in wt and *tub2-F265L* strains, as indicated. Two-tailed Mann–Whitney test: ***P<0.001; *n*>33. (D) Nuclear positioning in control and mutant cells. Cells grown at 30°C and 37°C were stained with Hoechst, and numbers and positions of nuclei were determined by fluorescence microscopy. Chi-square test: ***P<0.001; *n*>150.

We next aimed to test whether the altered G2-M microtubule dynamics and impaired cell growth at 37°C observed with *tub2-F265L* strains was related to defects in cell cycle progression. Direct observation of the cells during exponential growth showed an accumulation of *tub2-F265L* cells with a large bud compared to controls (Fig. 3A), suggesting a delay in mitotic progression in *tub2-F265L* cells. During this stage, yeast cytoplasmic microtubule’s main function is to position the nucleus close to the bud neck, to align the spindle along the mother-bud axis and to pull it through the neck (Markus et al., 2012a). We therefore examined mitotic spindle orientation in *tub2-F265L* cells during vegetative growth. As shown in Fig. 3B, defective orientation of the G2-M spindle relative to the mother-bud axis was observed in *tub2-F265L* cells (the mean spindle orientation angle was 43° in *tub2-F265L* cells compared to 25.7° in wt cells), and the distance between the nucleus and the bud neck was greater [mean distance from the proximal spindle pole to the bud neck: 1.63 µm in *tub2-F265L* cells versus 0.71 µm in wt cells (*n*>30, Fig. 3C)]. At restrictive temperatures, spindle misorientation was exacerbated, with 92.6% of *tub2-F265L* cells containing abnormally oriented spindles in the mother cells, compared to just 36% of wt cells (Fig. 3B). Thus, expression of F265L β-tubulin in yeast cells resulted in impaired spindle positioning that might explain the delay in initiating G2-M and the reduced viability observed at 37°C. We next used Hoechst staining to test whether nuclear partitioning occurs normally. As expected, mis-positioning of the nucleus in the mother cells, away from the neck, was observed in *tub2-F265L* cells compared to wt cells (Fig. 3D). However, very few *tub2-F265L* cells retained both nuclei in the mother cell (Fig. 3D). This result shows that, although the mitotic spindle orientation and position in the *tub2-F265L* mutant was abnormal, relatively few *tub2-F265L* cells executed abnormal anaphase to produce binucleate budded cells. Thus, accurate nuclear segregation may occur as a consequence of the dynein pathway that powers spindle movement (Badin-Larcon et al., 2004; Markus et al., 2012b).

### F265L mutation impairs recruitment of Bim1 (yeast EB1) at microtubule tips

**Fig. 4.**
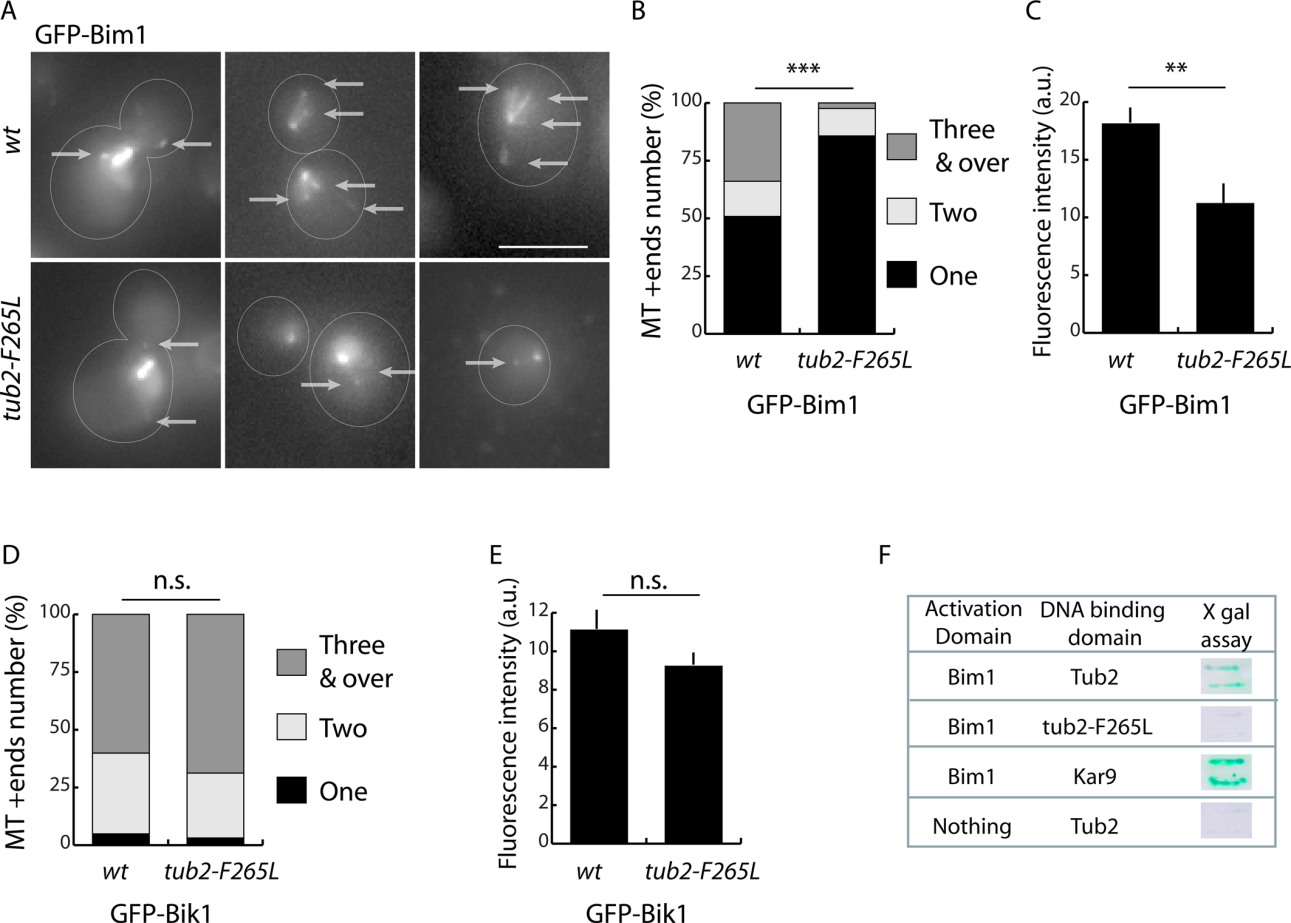
**Reduced binding of Bim1 (yeast EB1) to mutated F265L β-tubulin.** (A) Images of representative wt or *tub2-F265L* cells expressing GFP-Bim1 from its native promoter. Arrows indicate the plus-ends of cytoplasmic microtubules. Scale bar: 5 μm. (B–E) Quantitative analysis of fluorescence staining of GFP-Bim1 (B,C) and GFP-Bik1 (D,E) at the plus-ends of cytoplasmic microtubules. (B,D) The number of visually detected microtubule plus-ends was counted in wt and *tub2-F265L* strains. Chi-square test: ***P<0.001; n.s., not significant. (B) wt, *n*=59; *tub2-F265L*, *n*=43; (D) wt, *n*=33; *tub2-F265L*, *n*=31 cells. (C,E) Intensity of GFP-Bim1 (C) and GFP-Bik1 (E) at most fluorescent microtubule tip per cell. Two-tailed Mann–Whitney test: **P<0.01. (C) wt, *n*=138; *tub2-F265L*, *n*=48; (E) wt, *n*=90; *tub2-F265L*, *n*=81 microtubules plus-ends. Error bars correspond to s.e.m. (F) Two-hybrid interaction between Bim1 (fused to the GAL4 activation domain) and wt or F265L β-tubulin or a known interactor, Kar9, fused to the LexA DNA-binding domain. β-galactosidase activity was detected on filters to identify interactions.

Similar defective spindle orientation and nuclear positioning in mother cells have been reported in situations where the actin/Kar9 pathway is impaired (Miller and Rose, 1998). Bim1 is a key player in this pathway (Hwang et al., 2003; Lee et al., 2000; Miller et al., 2000). Hence, we examined Bim1 association with microtubule tips in the *tub2-F265L* strain. To do so, we used fluorescent fusion GFP-Bim1 and live-cell microscopy to observe and quantify Bim1 (Tirnauer et al., 1999). In wt cells, several microtubule ends were labeled with GFP-Bim1 (Fig. 4A) (Tirnauer et al., 1999), but we surprisingly found that microtubule ends labeled with GFP-Bim1 were barely detectable in *tub2-F265L* cells. Quantitative analysis revealed the extent of the reduction in the number of GFP-Bim1-stained microtubule tips (14.3% of cells with more than one plus-end labeled in *tub2-F265L* compared to 49.1% in wt, Fig. 4B). Furthermore, in *tub2-F265L* cells the Bim1 cap formed at microtubule ends displayed reduced fluorescence intensity compared with in wt cells (18.1 in wt compared to 11.2 in *tub2-F265L*, Fig. 4C).

These findings contrast starkly with the absence of any significant differences in microtubule number and GFP-Bik1 fluorescence intensity at microtubule plus-ends between wt and *tub2-F265L* cells (Fig. 4D,E).

We next investigated whether Bim1 could interact with F265L β-tubulin in a yeast two-hybrid test. As expected from published data (Lee et al., 2000; Schwartz et al., 1997), Bim1 interacted with wt β-tubulin (Tub2) and Kar9 in two-hybrid experiments (Fig. 4F). In contrast, interaction between Bim1 and F265L β-tubulin was barely detectable; reaching only the same levels as observed when no bait was added (Fig. 4F).

Taken together, these results indicate major specific defects in the interaction between Bim1 and F265L β-tubulin in two-hybrid experiments, and with microtubule ends in *tub2-F265L* cells *in vivo*.

The F265L mutation is located distal from the microtubule binding site described for the fission yeast EB (Mal3) (Maurer et al., 2012) and budding yeast Bim1 (Howes et al., 2017). This raises the question of the mechanism whereby the mutation perturbs Bim1 binding. Several possibilities may be envisioned: (i) the mutation might impair the microtubule association of another protein that might be needed for recruiting Bim1 to microtubules tips; (ii) a reduction in the size of the GTP cap at microtubule tips may reduce Bim1 comets; (iii) the mutation may cause allosteric disruption of the Bim1-binding site at the microtubule plus-end. The first of these potential explanations is argued against by *in vitro* data showing that most of the +Tips proteins are recruited to microtubules plus-ends via EB1 and Bim1 (Blake-Hodek et al., 2010; Duellberg et al., 2014). In addition, it would imply that an unknown partner also supports the two-hybrid interaction of Gal4-Bim1 with LexA-Tub2-F265L β-tubulin in the nucleoplasma.

End-binding proteins of the EB1 family bind to the GTP-cap (Duellberg et al., 2016; Maurer et al., 2012). If the second hypothesis were true, and as a reduced GTP cap size is correlated with a reduced microtubule growth rate (Rickman et al., 2017), we should expect a decrease in the growth rate of the mutated microtubules. In our experiments, the growth rates of F265L or wt microtubules are not different. *In vitro* studies have shown that lowering EB1 concentration increases time spent in pause (Maurer et al., 2014) which is consistent with our observation of longer pausing time in the *tub2-F264L* strain. Finally, in budding yeast, increases in time spent in pause have also been demonstrated in a strain deleted for *BIM1* (Tirnauer et al., 1999).

Hence, our data support the third hypothesis with an allosteric effect of the mutation reducing Bim1 affinity for F265L microtubule tips.

In mammalian cells, EB1 is apparently crucial for recruitment of a large number of proteins to microtubules ends, including the dynactin subunit p150 Glued and CLIP170. These two proteins are essential for dynein activity (Coquelle et al., 2002; Duellberg et al., 2014; Lansbergen and Akhmanova, 2006).

If our observations in yeast apply to mammalian tubulin, microtubule tip complexes might be reduced in neurons, impairing neuron migration or orientation of neural mitotic spindles (Tsai et al., 2007; Zhong and Chia, 2008). This study identifies yeast EB1 as a possible molecular target of the F265L β-tubulin mutation and offers new clues as to the mechanisms through which this mutation may interfere with human cortex development.

## MATERIALS AND METHODS

### Yeast strains and plasmids

Strains used in this study were isogenic to BY4743: *MAT*a/*MAT*α; *ura3*Δ0/*ura3*Δ0; *leu2*Δ0/*leu2*Δ0; *his3*Δ1/*his3*Δ1; *met15*Δ0/*MET15*; *LYS2*/*lys2*Δ0 for diploids, and BY4742; *MAT*α; *ura3*Δ0; *leu2*Δ0; *his3*Δ1; *lys2*Δ0 for haploids (provided by euroscarf, http://www.euroscarf.de). The *tub2-F265L*/*TUB2* (*MAT*a/*MAT*α; *ura3*Δ0/*ura3*Δ0; *leu2*Δ0/*leu2*Δ0; *his3*Δ1/*his3*Δ1; *met15*Δ0/*MET15*; *LYS2*/*lys2*Δ0 *TUB2*/*tub2-F265L*::*LEU2*) strain was obtained by a one-step replacement with plasmid pLEU2-tub2-F265L (see below). After preparing genomic DNA, confirmation by PCR and sequencing of the relevant region, three independent heterozygous diploids were isolated. The *tub2-F265L*/*TUB2* was sporulated and dissected for tetrad analysis. Three independent *tub2-F265L*-homozygous haploids were isolated.

The integrating plasmid pLEU2-tub2-F265L was constructed by a three-step process. First, the *TUB2* ORF with 1 kpb of downstream sequences was PCR-amplified from genomic DNA and cloned in pCR2.1 (Invitrogen) to generate pCR2.1-TUB2. Then, the *LEU2* locus was inserted 120 bp away from the *TUB2* stop codon at the *BsiW*I site. The F265L mutation was inserted *in situ* using the ATCC strategy and following primers (3′TCCCATTCCCACGTTTACATTTATTCATGGTCGGC, anti-sense 3′GCCGACCATGAATAAATGTAAACGTGGGAATGGGA).

pGFP-Bim1 (Tirnauer et al., 1999) and pGFP-Bik1 (Lin et al., 2001) were kindly provided by D. Pellman (Dana-Farber Cancer Institute, Boston, Massachusetts). For the two-hybrid experiments, the *KAR9*, *TUB2* and *tub2-F265L* genes, from genomic DNA, pCR2.1-TUB2 and pLEU2-tub2-F265L, respectively, were cloned into the pLexA vector (Addgene) to produce fusion proteins with the DNA-binding domain of LexA at the N-terminus. Bim1 genomic DNA was cloned into the pGADT7 vector (Invitrogen) to produce a fusion protein with the GAL4 activating domain at the N-terminus.

### Cell growth and cytological analyses

For growth tests on plates, fresh overnight cultures of wt and *tub2-F265L* (three different haploid clones for each strain) were monitored based on their OD600, and serial dilutions were spotted onto YPD plates with or without 30 µg/ml benomyl. Plates were incubated for 2 days at 30°C. To assess cell growth in liquid medium, fresh cultures were diluted to OD600=0.1, and cultured for several hours at 30°C or 37°C. All growth tests were performed with three independent clones. Cell morphologies (unbudded, small-budded and large-budded) were analyzed under optical microscopy. Cells with a bud size smaller than 1/3 of the mother cell size were considered small-budded cells. Cells with a bud size equal to or larger than 1/3 of the mother cell size were considered large-budded cells. The positions and the number of nuclei in large-budded cells were determined by staining with Hoechst 33258 (Sigma-Aldrich). The location of the spindle poles and spindle orientation was determined by overlaying images of GFP-Bim1 fusion fluorescence onto phase-contrast images of the corresponding cells. Spindle orientation was measured in all cells in the population containing a G2-M spindle with a pole-to-pole length of 0.8–1.2 μm, as described (DeZwaan et al., 1997). To study microtubule after benomyl treatment, overnight precultures of GFP-Bik1 transformed strains were diluted fourfold in SC-U, grown at 30°C for 2 h before addition of 30 µg/ml benomyl for 1 h and fix by adding PFA diluted to 4% directly in culture medium for 1 h.

### Microscopy and image analysis

Cell imaging was performed on a Zeiss Axiovert microscope equipped with a Cool Snap ES CCD camera (Ropper Scientific). All images were captured using 2×2 binning and 17 sequential z-axes collected at 0.3 μm, the exposure time varied between constructions. For microtubule dynamics measurements: maximum intensity projection images of cells bearing GFP-Bik1 fusions were used to visualize microtubules. Microtubule length at each time point was measured manually on maximum intensity projections, and dynamic parameters were calculated as described (Kosco et al., 2001), using an in-house Visual Basic macro embedded in a Microsoft Excel datasheet (Caudron et al., 2008). All image acquisitions were performed using MetaMorph software (Universal Imaging).
